# Society Proceedings

**Published:** 1902-04

**Authors:** 


### SOCIETY PROCEEDINGS.

### OFFICIAL REPORT OF THE PROCEEDINGS OF THE FOURTEENTH ANNUAL MEETING OF THE IOWA STATE VETERINARY MEDICAL ASSOCIATION.

*Held at Des Moines, Iowa, February* 11 *and* 12, 1902

FIRST DAY—*February* 11*th.*

*Morning Session.* The meeting was called to order at 9.45 o'clock, in Parlor I, Savery Hotel, by the President, Dr. P. O. Koto, of Forest City.

The Secretary announced that a system of card registration had been substituted for roll-call.

The Secretary read the minutes of the thirteenth annual meeting, which were approved as read.

The President delivered his address.

The Secretary read his report, as follows:

SECRETARY'S REPORT.

MR. PRESIDENT AND MEMBERS: It is by reason of the removal of Dr. John E. Brown from Iowa, and his consequent resignation from the office of Secretary of this Association, that I now make the report of the Secretary. The impaired health of Mrs. Brown necessitated the removal of Dr. Brown and family to a more equable climate. Chattanooga, Tennessee, was chosen as the most eligible place of residence, and it is in that city that the family now have their home. I have not the slightest doubt that I give expression to the feeling of all of you when I say that I greatly regret that it has been necessary for Dr. Brown to remove from Iowa and to sever his connection with this Association as an active member and as its Secretary. For eight years he served as Secretary and Treasurer of the Iowa State Veterinary Medical Association. During all of that time his services were so generously and unselfishly devoted to the interests of the organization that he won a warm place in the hearts of all its members and has always merited and received their praise. The present standing of this organization is due in a large measure to Dr. Brown's well-directed efforts. It is no easy task to take up and continue satisfactorily a work that has so prospered in the hands of the one who relinquishes it. It was, therefore, with reluctance that I assumed the duties of Secretary pro tempore last October, at the request of the President, Dr. P. O. Koto.

Owing to the fact that Dr. Brown had a considerable part of the books and papers of the Association packed with his goods, and that the goods were delayed in shipment to Chattanooga, the part of the material of the Association which was useful in carrying forward the work of the Secretary did not reach me until December 9th last, and, as a consequence, the work of getting up the programme and arranging for the meeting was delayed. For that reason the meeting is held later this year than is customary.

On November 9th I sent out to each member a circular letter asking for contributions to the programme of this meeting. As this was done in advance of receiving the list of members, and, as I had to depend upon my memory of who were and who were not members in mailing the letters, twenty-six members were missed. These members were reached by a subsequent communication after the list of members came into my hands.

After receiving the books containing the accounts with the members I sent to each member a statement of his account. This was done December 14th. In order to make more effective the attempt at collection I had a new form printed. The upper half of this form has printed upon it the directions to the members concerning the remittance and the article of the By-laws which covers the matter of membership fees and dues. The lower half contains the blank form for statement of account. These two halves are separated by perforations, so that the lower half may be torn off and sent with the remittance, to be receipted by the Treasurer, returned to the member, and held by him as his receipt. I believe all of the members have been given an opportunity to see this form.

On January 1st I found that the responses to my request for papers had not been liberal enough to insure a well-filled programme, so at that time I sent out a personal request for a paper or report of a case to a number of members whom I thought would give their help. From this appeal I got enough favorable replies to make the programme what you see to-day.

I would observe at this point that the majority of the members are not ready enough to supply a paper or report to the programme. This keeps the Secretary in a constant state of anxiety lest the time for the meeting arrive without an adequate programme. You are all aware that the meeting would be an absolute failure but for the papers and reports submitted. In fact, without these it would hardly be necessary to have a meeting at all. It could in any event be only a business meeting. It is equally well known that the Secretary without the co-operation of the members is a hopeless and useless part of the machinery of the Association. Why, then, should it be made for him such a difficult task to secure contributions to the programme? Will you not wake up to a proper realization of the urgency and importance of replying to these requests of your Secretary, and even before this meeting comes to an end may the Secretary not have promise of a paper or report of a case from a goodly number of you? You can decide in the period which intervenes between now and the next meeting what subject you will write upon.

On looking up the records it was found that twelve veterinarians still residing in the State were once members, but have been suspended for non-payment of dues. A letter, with which was enclosed a statement of dues owing at time of suspension, was sent to each of these, asking him to pay his arrears and apply for re-instatement. A few replies were received, which will be referred to at another time.

To each of several members who have left the State, but who are in arrears in their dues, a letter was sent, with enclosure of a statement of arrears, requesting that these arrears be paid and that application be made for re-instatement to membership. The responses to these requests will be referred to at another time.

On January 7, 1902, as near as I was able to learn, there were in the State fifty-seven graduated veterinarians who are not members of the Iowa State Veterinary Medical Association. A few of these had been members at one time, but had resigned. To each of these was sent a circular letter urging him to make application for membership. Accompanying this was sent a blank application form. I thought best to get out a new form for this purpose. The old form was very unhandy and somewhat antiquated, and the supply had been pretty well exhausted. In this new form the printing is all on one page, and the slip is of such size that it can easily be filed without folding. The entire substance will in this way be in such form that it can be taken in at a glance. It is divided into four sections: First, there is set forth the article of the constitution governing the matter of membership, together with some instructions. Second, there is a blank application form. Third, there is a blank for report of Board of Censors. Fourth, there is a blank form for report of the Secretary on the result of the ballot of the Association upon the candidate.

To this date eight applications for membership have been received by the Secretary. This number will, I hope, be greatly increased during this meeting. I would observe here that it is rather surprising that more veterinarians are not awake to the great advantages of membership in our State organization. A member not only has the consciousness that he is strengthening himself by uniting with others in this way, but also the consciousness that he and his associates are elevating the profession of veterinary medicine and bringing it to the attention of the laity, and in this way commanding their commendation of our noble pursuit. A veterinarian cannot do better for himself or for his profession than to carry an honorable membership in a State veterinary organization. It is to be hoped that all the members will unite in an effort to bring every eligible veterinarian in the State within the bounds of our Association.

On January 17th the programme and the announcement for this meeting were mailed to each veterinarian in the State, whether a member or not, and to each honorary member; also, a copy of each was sent to each of several prominent newspapers and agricultural journals, with the request that they be used as subject-matter for the news columns. This request was complied with in every case that I was able to trace.

In addition to this a general correspondence has been kept up with various members and others when occasion required. I would like to say here that the Secretary would be pleased to have a letter from each member and veterinarian not a member at least once a year. I regret to say that this pleasure has not been had during my incumbency to date.

I would call your attention to the following statement:

| | | |
|---|---|---|
| Veterinarians in Iowa | | 154 |
| Resigned from membership in the I. S. V. M. A. (in State, 3; out of State, 4 (?) | 7 | |
| Suspended from membership (in State, 17; out of State, 22) | 39 | |
| Honorary members (in State, 1; out of State, 9) | 10 | |
| Active members (in State, 76; out of State, 22) | 98 | |
| Active members in good standing | | 62 |
| (*a*) With dues paid up to and including 1902 | 27 | |
| (*b*) With dues paid up to and including 1901 | 21 | |
| (*c*) With dues paid up to and including 1900 | 14 | |
| Members not in good standing | | 36 |
| (*a*) Only three years in arrears | 7 | |
| (*b*) Only four years in arrears | 2 | |
| (*c*) Only five years in arrears | 4 | |
| (*d*) Only six years in arrears | 6 | |
| (*e*) Only seven years in arrears | 7 | |
| (*f*) Only eight years in arrears | 10 | |

As the sum required for yearly dues is only one dollar, it is no small wonder that so many would allow themselves to fall into delinquency. I am glad to say, however, that my attempts at collection have been quite successful thus far. It is probable that if statements were regularly sent the members would have their attention directed to the fact that they really owe the Association something, and that they would be more prompt in payment. This plan should be given a thorough test.

I would refer briefly to Article IV. of the By-laws, as amended at the 1897 meeting, which reads as follows: "Each member, on being admitted to membership, shall pay the sum of two dollars, and shall annually thereafter pay one dollar in advance to the Association; and any member in arrears more than two years shall be suspended until said arrears are paid."

It seems to me that the words "shall be suspended" in themselves work a suspension when a member has fallen into arrears more than two years; or, at least, these words make suspension compulsory on the part of the Association. If a member were suspended when he comes into arrears as much as three years, and it were generally known that he would be so suspended, I doubt if many would allow themselves to become delinquent to such an extent. At all events, if the member were promptly suspended at that time his dues would not accrue any further, and he would never be called upon to pay more than three dollars back dues in order to secure reinstatement. Under such conditions he would be much more likely to pay up arrears and ask for reinstatement than if his dues were allowed to accrue for five or six years or longer. This is a matter upon which the Association should take some action at this meeting.

Following is a statement of the finances covering the period of my incumbency, from October 1, 1901, to February 10, 1902:

| | | |
|---|---|---|
| *Receipts.* | | |
| Cash from J. E. Brown | $66 57 | |
| Refund, American Express Company | 1 35 | |
| Cash for dues for 1902 | 26 00 | |
| Cash for back dues | 35 75 | |
| Membership fees | 12 00 | |
| | | $141 67 |
| *Disbursements.* | | |
| Express | $2 00 | |
| Stationery | 17 90 | |
| Blanks | 3 50 | |
| Stamps | 15 50 | |
| Telegrams | 90 | |
| Announcements | 1 50 | |
| Programmes | 3 00 | |
| Badges and express | 14 20 | |
| Secretary's expenses | 14 62 | |
| Cards | 2 25 | |
| | | 75 37 |
| Balance handed to the Treasurer | | $66 30 |

I desire to return my thanks to all those who have so willingly assisted the Secretary in his work during the preparation for this meeting.

Respectfully submitted,

JOHN J. REPP,
Secretary pro tem.

### Treasurer's Report.

The Treasurer submitted the following report:

1. For John E. Brown.—*Receipts.*

| | | |
|---|---|---|
| Cash on hand from twelfth annual meeting | $78 80 | |
| Cash for dues for thirteenth annual meeting | 38 50 | |
| Cash for membership fees | 8 00 | |
| | | $125 30 |
| *Disbursements.* | | |
| Presentation of canes | $33 55 | |
| Hall rent, thirteenth annual meeting | 5 00 | |
| Secretary's expenses | 10 38 | |
| Postage | 1 00 | |
| Stationery | 1 25 | |
| Paper | 35 | |
| Stenography | 1 25 | |
| Express, Oskaloosa to Ames | 1 10 | |
| Express, Waynesville to Ames | 1 35 | |
| Letter-heads | 3 50 | |
| | | 58 73 |
| Balance to John J. Repp | | $66 57 |

2. For Self.—*Receipts.*

| | | |
|---|---|---|
| Cash from John E. Brown | $66 57 | |
| Refund, American Express Company | 1 35 | |
| Cash for dues for fourteenth annual meeting | 26 00 | |
| Cash for back dues | 35 75 | |
| Cash for membership fees | 12 00 | |
| | | $141 67 |
| *Disbursements.* | | |
| Express | $ 2 00 | |
| Stationery | 17 90 | |
| Blanks | 3 50 | |
| Stamps | 15 50 | |
| Telegrams | 90 | |
| Announcements | 1 50 | |
| Programmes | 3 00 | |
| Badges and express | 14 20 | |
| Secretary's expense | 14 62 | |
| Cards | 2 25 | |
| | | 75 37 |
| Balance in Treasurer's hands | | $66 30 |

Respectfully submitted,

John J. Repp,
Treasurer pro tem.

The following committee was appointed to audit the Treasurer's accounts: Dr. H. L. Stewart, Dr. C. A. Clinton, Dr. George M. Walrod.

The Auditing Committee made the following report:

### Report of Auditing Committee.

Des Moines, Iowa, February 11, 1902.

We, the Auditing Committee for the fourteenth annual meeting of the Iowa State Veterinary Medical Association, hereby certify that we have examined the above account of the Treasurer, and that we find it correct.

George M. Walrod,
C. A. Clinton,
H. L. Stewart,
Auditing Committee.

By vote of the Association the report was adopted.

The Secretary read communications from various members and others. Of especial interest was the following from Dr. John E. Brown, of Chattanooga, Tenn., former Secretary of the Association:

Chattanooga, Tenn., January 28, 1902.

*To My Dear Old Friends and Members of the Iowa State Veterinary Medical Association—*

Gentlemen: The announcement and most excellent programme of the next annual meeting of the Association, to be held on February 11th and 12th, are before me.

I can scarcely bring myself to realize that my position, geographically, is so remote from you that henceforth I must be deprived of the exceeding pleasure of attending these meetings. That I can no longer be, in person, one of you with whom it was my privilege to mingle so pleasantly and so profitably for so many years is to me a matter of very deep regret.

Side by side we have worked together for the advancement of the various interests of our profession, without a hitch, and I cherish the thought that after all these years I can think of each and every member as a warm personal friend as well as co-operator in association matters. While it is true I am temporarily, through force of circumstances, out of practice, I am still, in the true sense of the term, a veterinarian, and your interests are mine. I hope to live to see the day when the veterinary profession will be honored and appreciated just as much as any other, and will then rejoice as heartily as anyone. Simply treating the sick and injured horses, cattle, and other animals is not the highest calling of the profession. Thus far it is looked upon more as a trade with a financial basis of valuation. Just as soon as the people learn that veterinarians stand for higher sanitary conditions—conditions which mean better health and more happiness to the human race as well as to the lower animals—and the veterinary profession proves that its members are true, educated, scientific sanitarians, then, but not until then, will the profession enjoy the high respect, confidence, and power, socially and professionally, that every progressive veterinarian knows it should. Iowa in the years past has made a beautiful start in the right direction in this respect. She has set a noteworthy pace for her sister States to follow. I do not fear that in the years to come she will fall behind the pace she has already set.

The North and West are far better suited to veterinary practice than the South. The ratio of opportunities for veterinarians in the North and the South is about ten to one in favor of the former. The stock interests here are undeveloped. As a rule, the stock seen through the country is very inferior, both in quantity and in quality, as compared with the Northern and Western States. There is a gradual improvement in the stock interests throughout the South, and sooner or later a fertile field for veterinarians will open.

We as a family are greatly enjoying the modified winter temperature, and are passing the winter with decidedly improved health. While we do and long will miss the familiar faces and cheery "hellos" of our Iowa friends, these changes go a long way toward making us like our new home "way down in Dixie."

And now, my friends, while it becomes my unpleasant duty to present my resignation as Secretary and active worker in the Iowa State Veterinary Medical Association, in spirit I will still be with you, especially on February 11th and 12th. I shall cherish the kindest remembrances of the Association, and will ever rejoice in any and all of its achievements.

In a parting word I would, if I could, inspire each Iowa veterinarian with a still greater interest in the State Association and loyalty to its interests. Combine your efforts, concentrate your forces, permit naught but harmony in your ranks, and your progress will mean success; also, let me bespeak for my successor in office the same loyal, courteous treatment you have always accorded to me.

Long may you prosper. Remember my latch-string is out to any member who may ever happen to journey this way.

Ever yours, with best wishes,

J. E. Brown.

Dr. Gibson moved that a special committee of three be appointed by the President to draft resolutions in regard to Dr. John E. Brown, former Secretary of the Association. This motion prevailed.

The President appointed the following committee: Dr. J. I. Gibson, Dr. John J. Repp, Dr. George A. Scott.

The Board of Censors reported favorably upon the following applicants for membership: Dr. G. L. Buffington, Baxter; Dr. N. A. Kippen, Riceville; Dr. F. F. Parker, Oskaloosa; Dr. J. S. Potter, Iowa City; Dr. John V. Jewell, Le Mars.

The President appointed as tellers Dr. S. H. Kingery and Dr. C. E. Stewart.

On ballot by the Association the following were elected to active membership: Dr. G. L. Buffington, Dr. N. A. Kippen, Dr. F. F. Parker, Dr. J. S. Potter.

On motion, the ballot on the application of Dr. John V. Jewell was postponed indefinitely because no one was present who knew Dr. Jewell well enough to inform the members fully in regard to him.

Adjournment was taken to 1.30 P.M.

*Afternoon Session.* Meeting called to order by President Koto at 1.30 o'clock.

New business was taken up.

The Secretary moved that all members three years or more in arrears in dues be suspended until all back dues are paid. Seconded by Dr. Kingery. The Secretary then explained that this motion was in accordance with the provision of Article IV. of the By-laws of the Association.

Dr. Walrod moved to amend the motion so as to exclude from suspension all those who would pay their dues before adjournment of the meeting for the afternoon. This amendment was duly seconded, put to a vote, and carried.

On vote, the motion as amended was adopted. The Secretary then read a list of thirty-six members to whom this action would apply.

Dr. Clinton moved to instruct the Secretary to send a registered communication to each member suspended, notifying him of his suspension and stating that all back dues must be paid before he could be reinstated. This was seconded, put to a vote, and adopted.

Dr. Kingery moved that suspended members who desire reinstatement must, after paying back dues, apply for reinstatement, and that the application must be submitted to a vote of the Association by ballot; seconded by Dr. Clinton, put to a vote, and carried.

On motion, Dr. John E. Brown, Chattanooga, Tenn., and Dr. H. E. Titus, Lafayette, Ind., both active members with dues paid up, were elected to honorary membership for the period during which they reside outside of the State.

In the absence of the author, the Secretary read Dr. Hamilton's report of "A Case of Anthrax in a Horse."[1]

As Dr. Simpson was not present, his report on "Chronic Atrophic Orchitis in a Bull"[2] was read by Dr. Kingery.

The Board of Censors reported favorably upon the following applications for membership: Dr. A. B. Wilmoth, Des Moines; Dr. A. A. Adamson, Newton; Dr. T. F. McEvers, Colfax.

The President appointed as tellers Dr. C. E. Stewart and Dr. P. Malcolm.

On ballot of the Association the following were elected to active membership: Dr. A. B. Wilmoth, Dr. A. A. Adamson, and Dr. T. F. McEvers.

The Secretary moved to reinstate Dr. W. A. McClanahan, who had been suspended for non-payment of dues, to active membership.

On ballot of the Association Dr. McClanahan was reinstated. At this point, on motion, the Association went into secret session.

Dr. Repp moved that the non-graduate practitioners be given no more recognition than any other visitors; seconded.

After some discussion, Dr. Austin moved to amend so as to extend an invitation to all except registered non-graduates to attend the meetings. This was fully debated, and, on vote, was defeated.

The original motion was then voted upon and carried.

At 6.30, on motion, adjournment was taken to 8 P.M.

*Evening Session.* Called to order by President Koto at 8.30 o'clock.

The President appointed Dr. J. I. Gibson and Dr. George A. Scott to fill vacancies on Committee on Resolutions due to the absence of Dr. G. E. Noble and Dr. William Hamilton.

The report of the Committee on Sanitation was then made by the Chairman, Dr. T. A. Shipley.

(To be continued.)

### INDIANA STATE VETERINARY ASSOCIATION.

THIS Association held its twenty-sixth semi-annual meeting in the State House at Indianapolis, Ind., January 9, 1902. The meeting was called to order at 1 P.M. by the President, J. C. Rodgers, of Anderson, Ind. On motion, the regular order of business was suspended, all of the members repairing to a clinic-room previously prepared by the Programme Committee. The following cases and operations were witnessed by eighty gentlemen of the profession: Spaying bitches, by Dr. G. H. Roberts and Dr. J. W. Klotz; median neurectomy, by Dr. P. O. O'Rear; peroneal neurectomy, by Dr. Frank W. Brewer; cryptorchid operation and ovariotomy, by Dr. J. W. Klotz; high plantar neurectomy, by Dr. E. H. Pritchard; and tenotomy, by Dr. J. W. Klotz. All these operations were performed under anæsthesia.

Dr. R. A. Craig exhibited some very fine specimens of glanders, nasal chambers, and lungs. There was a very interesting case of valvular insufficiency and hypertrophy of the heart in a gray gelding belonging to the Indianapolis Fire Department.

The meeting was called to order by the President at 7.30 P.M. After the reading of reports of the several committees the following officers were elected for the present year:

W. F. Myers, President; W. B. Wallace, Vice-President; O. L. Boor, Treasurer; G. H. Roberts, Secretary.

Some very interesting papers followed: Dr. F. W. Anderman read a paper on "Cerebro-spinal Meningitis," which was prepared as the result of an epizoötic outbreak. This paper was well discussed. Dr. F. W. Brewer read a very valuable paper on "Neurectomy," which showed that the doctor was conversant with this subject. Dr. L. C. Hoover being absent, his paper on "Laminitis" was read by Dr. P. O. O'Rear. A lengthy discussion followed. Dr. O. L. Boor read a paper on "Some Complications of Influenza." The complication that was generally discussed was nephritis.

The following gentlemen were present: Drs. O. L. Boor, Muncie; J. O. Greeson, Kokomo; J. W. Klotz, Noblesville; W. F. Meyers, Fort Wayne; P. O. O'Rear, Indianapolis; James Crail, Shelbyville; J. B. Kinter, Danville; C. P. Wilson, Greenfield; Robert Harper, Indianapolis; G. H. Roberts, Indianapolis; John E. Pritchard, Indianapolis; J. M. Pattison, Connersville; J. C. Rodger, Anderson; and I. E. Scripture, Frankfort.

The following new members were admitted: J. D. Reynierson, Thorntown; J. G. Highaway, Ladoga; E. C. Carpenter, Bloomington; W. B. Wallace, Marion; E. N. Stout, Stockwell; R. A. Carig, Lafayette; H. E. Titus, Lafayette; D. C. Smith, Frankfort; N. B. Combs, Mulberry; F. W. Brewer, Southport; Parker Justice, Delphi; and E. M. Bronson, Portland.

The Association adjourned to meet in September.

GEORGE H. ROBERTS,
Secretary.

### MINNESOTA STATE VETERINARY MEDICAL ASSOCIATION.

THE tenth semi-annual meeting of the Association was called to order by the President, Dr. J. N. Gould, at the Merchants' Hotel, in St. Paul, at 2 P.M., January 15, 1902.

The following members were present and responded to roll-call: Drs. C. C. Lyford, M. H. Reynolds, S. D. Brimhall, R. Price, L. Hay, K. J. McKenzie, S. H. Ward, G. A. Dallamore, J. G. Annand, J. N. Gould, George McGillivary, J. W. Gould, B. A. Pomeroy, H. C. Lyons, J. S. Butler, H. C. Peters, M. J. Sexton, F. H. Farmer, E. T. Frank, C. T. Eckles, J. P

Foster, R. La Pointe, John McKay, J. W. Cook, D. M. McDonald, J. M. Lambert, Oscar Rydell, F. A. Illstrup, J. J. Findley, George Ed. Leech, and R. H. Jerner.

The Treasurer's report was read and accepted.

The following applicants were duly elected to membership: Dr. R. K. Jerner (O. V. C , '96), Chatfield, Minn.; Dr. Edward L. Kalb (O. V. C., '93), Rochester, Minn.; Dr. George Ed. Leech (C. V. C., '91), Winona, Minn.; Dr. J. J. Finlay (O. V. C., '88), Duluth, Minn.

Dr. D. M. McDonald reported for the Committee on Colleges. Dr. Hay read his report on recent veterinary literature pertaining to medicine, describing some interesting cases of poisoning by overdoses of sodium hyposulphite. Dr. Hay recommended the administration of potassium iodide in ounce doses a short time prior to parturition as a prophylactic measure to prevent parturient paresis.

Dr. Price next read his report on bacteriology. This report brought out a lively discussion on tetanus and antitoxins.

Dr. Annand's report on surgery came next, describing a new operation for the treatment of impervious urachus. Dr. Annand also discussed the treatment of indolent summer wounds; also the best method of overcoming spasmodic contraction of the os uteri.

Dr. Brimhall next reported for the Committee on Infectious Diseases, stating that there had been 1235 cases examined for glanders during the past year; 500 had been tested with mallein and 325 had been condemned and killed. There had been 9982 cattle tested for tuberculosis, of which 458 reacted and 315 had been killed. Hog cholera had not been prevalent. There had been 90 cases of actinomycosis reported, of which 20 cases were condemned as not fit for food purposes. Blackleg existed in four counties. Hemorrhagic septicæmia had been reported from several counties.

Dr. Reynolds next reported for the Committee on Legislation and Empirics. The doctor proposed a committee of three to take charge of the prosecution of quacks. On motion, a committee of three was appointed. The committee appointed was Drs. S. H. Ward, M. H. Reynolds, and J. S. Butler.

The election of officers then took place, and resulted as follows:

President, Dr. C. C. Lyford, of Minneapolis, Minn.; First Vice-President, Dr. E. T. Frank, of Warren, Minn.; Second Vice-President, Dr. J. P. Foster, of Selby, South Dakota; Secretary and Treasurer, Dr. K. J. McKenzie, of Northeld, Minn.; Trustees, Drs. J. W. Cook, H. C. Peters, and M. H. Reynolds.

On motion, a committee of three was appointed to draw up resolutions expressing our sympathies and feelings toward Dr. Youngberg, and also regarding the death of Dr. Huidekoper. The committee appointed was Drs. Brimhall, Frank, and Price.

### Resolutions.

We, the members of the Minnesota State Veterinary Medical Association, learn with sincere gratification of the markedly improved condition and hope for a speedy recovery of our esteemed colleague, Dr. A. Youngberg, of Lake Park, Minn.

*Resolved*, That a copy of the above be spread upon our minutes and that a copy of the same be sent to Dr. Youngberg by our Secretary.

Dr. E. T. Frank,
Dr. R. Price,
Dr. S. D. Brimhall,
Committee.

Whereas, the Minnesota State Veterinary Medical Association learns with regret of the decease of our most eminent and esteemed colleague, Dr. Rush Shippen Huidekoper; we feel that in his death the veterinary profession has lost a most brilliant and untiring worker; therefore, be it

*Resolved*, That we hereby express our appreciation of his unselfish life-work for his profession and our sorrow at his death.

Dr. Brimhall,
Dr. Frank,
Dr. Price,
Committee.

Adjournment for supper was taken.

After supper the members met at the State Experimental Station, where several cases were exhibited that had been operated upon at previous meetings, among which were two cases of capped hock that had been operated upon two years previous; they had proven quite a success. Drs. Reynolds and Peters operated for the repulsion of a fourth molar, performing Williams' operation, removing the outer plate of the alveolus. Dr. Lyford exhibited several cases sent over from his infirmary in Minneapolis.

Thursday forenoon was spent at the bacteriological laboratory of the State University, where the members were entertained by Dr. Westbrook, the bacteriologist, and Dr. S. D. Brimhall. The members' visit to the laboratory proved a profitable as well as an enjoyable forenoon.

Thursday afternoon the meeting was again called to order at the Merchants' Hotel, where the following paper was read: "Clinical Notes on Contagious Pneumonia," by Dr. S. H. Ward. The doctor reported thirty cases in one outbreak, of which number eight died. Autopsies were held and specimens sent to Dr. Westbrook, of the bacteriological laboratory, who confirmed the diagnosis. Dr. Ward's paper was quite lengthy and brought out a lively discussion. Dr. Peters read his paper on "Verminous Aneurism of the Mesenteric Artery the Starting-point of a Fatal Septicæmia." This paper was original, and proved Dr. Peters to be a student along the bacteriological line.

Dr. L. Hay next read his paper on "Eserine: Its Uses and Abuses." This paper brought out a spirited discussion on the uses of eserine and on colics in general.

Dr. Lyford read a paper on "Bursal Enlargements," exhibiting photographs of cases before and after being operated upon. This was a paper which had been read before the American Veterinary Medical Association, and was presented before our Association at the request of quite a number of the members interested in Dr. Lyford's radical surgery.

On motion, this Association guaranteed a fund of $500 to help entertain the American Veterinary Medical Association, providing their next meeting should be held in Minneapolis.

On motion, the President was empowered to appoint a committee of as many as he deemed necessary to help in entertaining the American Association.

On motion, a Press Committee was created, consisting of three, to look after the press work of the Society. The following constitute the committee as appointed: Drs. M. H. Reynolds, S. H. Ward, and K. J. McKenzie.

At this juncture Dr. S. D. Brimhall read a communication from the clerk of a court located in New York regarding the qualifications of a Dr. Smead now lecturing with the "Minnesota State Farmers' Institute."

On motion, the following was added to our By-laws: Article X, Code of Ethics, as embodied in the By-laws of the American Veterinary Medical Association, embracing also that it shall be considered a breach of ethics for any member of this Association to reveal to anyone not a member of this Association particular or specific treatment practised by a member of this Association for the encouragement and help of each other.

At this juncture President Lyford appointed the following committees: Colleges, Dr. H. C. Peters; Infectious Diseases, Dr. J. G. Annand; Bacteriology, Dr. S. D. Brimhall; Surgery, Dr. L. Hay; Medicines, Dr. R. Price; Legislation, Dr. S. H. Ward, Dr. M. H. Reynolds, Dr. J. S. Butler; Finances, Dr. W. Amos; Resolutions, Dr. S. D. Brimhall, Dr. E. T. Frank, Dr. Richard Price; Press, Dr. M. H. Reynolds, Dr. S. H. Ward, Dr. K. J. McKenzie.

The next meeting of our State Association with the American Association was discussed quite freely. No formal action was taken, but the opinion was freely expressed that it would be desirable to hold a very short business meeting, probably on the Monday afternoon preceding the meeting of the American Veterinary Medical Association, adjourning the summer meeting from July 1st to September 1st accordingly.

Dr. Reynolds urged the members of the State Association to assist him in building up a veterinary museum that would be a credit to the State, urging particularly that material should be selected with a view of teaching value and not as mere curiosities, each donor to be given full credit on the descriptive label or placard. Dr. Reynolds also suggested the advisability of having the regular meetings reported, and thought this could be arranged for without great expense.

The following resolution in regard to Dr. Smead, veterinarian to the State Farmers' Institute Corps, was introduced by the Resolution Committee previously instructed to do so:

WHEREAS, One known as Dr. Clarence B. Smead is posing in the State as a qualified veterinarian, and is known as the "State Farmers' Institute Veterinarian;" and

WHEREAS, We learn from reliable sources that he is not a graduate of any regular recognized authorized veterinary college; therefore, be it

*Resolved*, That this Association protest against his appearing under the title of "Doctor of Veterinary Medicine," or any other title, or be recognized in any way which will carry the impression that he is a qualified veterinarian.

S. D. BRIMHALL,
E. T. FRANK,
RICHARD PRICE,
Committee.

The resolution was adopted and the Secretary instructed to present the same to the Board of Control of the State Farmers' Institute, and also to Superintendent Gregg, of Lynn, Minn.

Dr. Price presented in writing a motion to change the date of the annual meeting from the second Tuesday to the Thursday following the second Tuesday in January. The question arose as to the validity of this action, but it was decided to call for a vote upon Dr. Price's motion to change the date of the meeting, and the motion being duly seconded was carried unanimously—"motion to change the date of the annual meeting" to the Thursday following the second Tuesday in January.

K. J. McKENZIE,
Secretary and Treasurer.

### ALLEGHENY COUNTY VETERINARY MEDICAL ASSOCIATION.

THIS young Association held well-attended monthly meetings during the winter and accomplished much good committee work, especially in efforts to adopt and establish a scale of fees or charges for professional visits and surgical operations. It was claimed some parties were making a practice of dividing fees with coachmen, and others were charging less than customary in this locality.

Drs. Gearhart, Richards, Spindler, and Waugh were appointed a Committee on Resolutions on the death of the late Dr. R. S. Huidekoper.

Drs. Boyd and Spindler presented excellent papers and reported interesting cases in practice.

Dr. A. Leteve, of Magee Pathological Institute, Mercy Hospital, delivered a very interesting and instructive lecture on the latest facts relating to tetanus, including serum therapy combinations, giving favorable results. Drs. McNeil, Meyer, and Waugh indulged in general discussion, and Dr. Leteve kindly answered many inquiries.

JAMES A. WAUGH, V. S.,
Secretary.

### KEYSTONE VETERINARY MEDICAL ASSOCIATION.

THE regular monthly meeting was held in their room, northeast corner of Broad and Filbert Streets, March 11, 1902, with the President, Dr. Rhoads, in the chair. Owing to the absence of the regular Secretary, Dr. Ranck, Dr. Hoskins was appointed Secretary pro tem.

On roll call the following members were found present: Drs. Rhoads, Lintz, Felton, Hoskins, James T. McAnulty, and of the profession Dr. H. . Cox.

The reading of the minutes of the preceding meeting was on motion dispensed with.

Dr. Hoskins, as the Committee on Milk, reported and presented to the Association one of the blanks that the committee purposes sending to all of the institutions of a public or semi-public character in the city of Philadelphia.

Dr. H. B. Cox's (graduate of the American Veterinary College) application for membership was received, being vouched for by Drs. James T. McAnulty and W. Horace Hoskins, and referred to the Board of Trustees. To fill vacancies on the same the President appointed Drs. Lintz and McAnulty. The Board having reported favorably on the application, his election followed, and the President introduced him to the members present.

Dr. Charles M. Lintz then gave a lengthy dissertation on the trotting-bred horse. In his remarks he placed the breeding of many of the best horses in the land, pointing out the wonderful influence exercised by "Hambletonian Ten" in the transmission of speed to his offspring.

Dr. Lintz was interrogated by all the members present, but his knowledge of the great racing trotting horses enabled him to answer intelligently all the interrogatories.

The meeting adjourned at 11 o'clock.

W. Horace Hoskins,
Secretary pro tem.

### NIAGARA AND ORLEANS COUNTY VETERINARY MEDICAL SOCIETY.

The semi-annual meeting of the above Society was called to order at Medina, March 18th, by the President, Dr. W. E. Stocking, in a few well chosen remarks.

The Secretary, Dr. Crowforth, read the minutes of the annual meeting. The question of legislation was discussed at length, the Society being a unit in opposition to any bill that would cause the profession to recede to the laws of 1886, or anything that would detract from the present high standard of required veterinary education in this State. The veterinary and medical professions are working hand-in-hand in relation to the protection of public health, and both should be placed on the same educational footing. It was reported that a number of veterinarians residing in Canada on the border are continually practising in this State without being registered according to the State law. After a lengthy discussion the report was laid on the table until next meeting.

Dr. James Martin was elected to membership. He is the senior member of the Society.

Roll call found every graduate in the two counties present.

The subject of parturition was taken up, and each member entered into the discussion with far greater ease than could be displayed on a living subject. Dr. Hunter, of Niagara Falls, and Dr. Kesler, of Holly, were appointed essayists for the next meeting, at Lockport, in August, 1902.

Anderson Crowforth,
Secretary.

### WISCONSIN SOCIETY OF VETERINARY GRADUATES.

The annual meeting of the Society assembled in the Capitol Building, at Madison, at 2 o'clock P.M., March 5, 1902. The meeting was called to order by the President, Dr. C. E. Evans.

Roll call. Those present were Drs. W. G. Clark, B. L. Fossee, A. H. Harting, R. S. Heere, J. T. Hernsheim, L. N. Jargo, G. Ed. Leech, E. A. McCullough, A. J. Nelson, F. J. Roub, E. D. Roberts, D. Roberts, and S. S. Snyder.

Visitors present were Drs. Geo. E. Allen, Fort Atkinson; M. H. Reynolds and S. D. Brimhall, of Minneapolis, Minn.

The Secretary's report was read and accepted.

The Treasurer's report was read and accepted.

It was moved and seconded that the Chair appoint a committee to draw resolutions on the death of Dr. C. H. Ormond. Carried. The President appointed Drs. D. Roberts, Hernsheim, and Snyder.

The terms of Drs. Clute and Leech on Committee on Legislation having expired, it was decided that the Chair appoint members to fill vacancies.

The application for membership of Dr. A. H. Beckwith, of Shullsburg, Wis., was reported favorably, and on motion Dr. Beckwith was declared elected.

Dr. G. Ed. Leech having removed to Winona, Minn., requested an honorary membership. On motion the application was granted.

Dr. F. J. Roub read a report of poisoning by sinapis nigra, which was discussed by Dr. D. Roberts. Dr. Beattie reported a similar case.

On motion a vote of thanks was tendered the essayist.

On motion the Society proceeded to the election of officers.

Dr. F. J. Roub was nominated for President. There being no other nomination, the Secretary was instructed to cast the ballot for Dr. Roub for President. The ballot was cast and Dr. Roub declared elected.

Dr. R. S. Heere was nominated for Vice-President. There being no other nomination, the Secretary was instructed to cast the ballot for Dr. Heere. The ballot was cast and Dr. Heere declared elected.

Dr. W. G. Clark was nominated for Secretary, but declined. Dr. S. Beattie was then nominated. There being no other nomination, the Secretary was instructed to cast the ballot for Dr. Beattie. The ballot was cast and Dr. Beattie was declared elected.

Dr. S. S. Snyder was unanimously re-elected as Treasurer.

Drs. B. L. Clark, A. J. Nelson, and H. B. Clute were nominated for Board of Censors. There being no other nominations, the Secretary was instructed to cast the ballot for the above gentlemen. The ballot was cast and they were declared elected.

On motion the Society adjourned to meet at 7.30 P.M.

W. G. Clark,
Secretary.

7.30 P.M. The President appointed Dr. R. H. Harrison to fill vacancy of Dr. C. H. Ormond on Revisionary Committee, and Dr. S. Beattie to fill vacancy of Dr. G. Ed. Leech on Committee on Legislation.

On motion Dr. Ormond was placed on honorary member roll.

Dr. W. G. Clark read a paper by Dr. J. M. O'Reilley on the use of eserine in the treatment of colic in the horse.

On motion discussion closed until next meeting, as essayist was absent.

Drs. M. H. Reynolds and S. D. Brimhall, of Minneapolis, Minn., were present in behalf of the American Veterinary Medical Association, and addressed the meeting, extending invitations to attend that meeting, to be held at Minneapolis, Minn., September 2 to 4, 1902.

It was moved and seconded that the Secretary send each member of our Association an invitation to attend the American Veterinary Medical Association meeting. Carried.

The veterinary laws of Wisconsin were discussed by several members, and it was moved and seconded that the Board of Legislators frame a bill for a State Board, this bill to be presented at our next meeting. Carried.

It was moved and seconded that a committee, consisting of Drs. Clute, D. Roberts, and Harrison, be appointed to furnish clinical material for our next meeting at Milwaukee. Carried.

Resolutions on the death of C. H. Ormond, of Milwaukee, were read as follows:

*Whereas*, It has pleased the Almighty to remove from our midst our esteemed member, Charles H. Ormond; and,

*Whereas*, The intimate relation and business intercourse with him have been most pleasant, make it befitting that we publicly record our appreciation of him; therefore, be it

*Resolved*, That in the loss of C. H. Ormond we lose a friend and valued member of our Association and profession; therefore, be it

*Resolved*, That the deep sympathy of this Association be extended to his relatives and friends; and be it further

*Resolved*, That a copy of these resolutions be forwarded to his relatives, spread upon our records, and published in the veterinary journals.

On motion the Society adjourned, to meet at Milwaukee subject to the call of the President and Secretary. S. BEATTIE,
Secretary.

